# The Lateral Tracking Control for the Intelligent Vehicle Based on Adaptive PID Neural Network

**DOI:** 10.3390/s17061244

**Published:** 2017-05-30

**Authors:** Gaining Han, Weiping Fu, Wen Wang, Zongsheng Wu

**Affiliations:** 1 School of Mechanical and Precision Instrument Engineering, Xi’an University of Technology, Xi’an 710048, China; han_gn@163.com (G.H.); wangwen@xaut.edu.cn (W.W.); wuzs2005@163.com (Z.W.); 2 School of Computer, Xianyang Normal University, Xianyang 712000, China

**Keywords:** intelligent vehicle, steer control, forgetting factor recursive least square, neural network, PID control, path tracing

## Abstract

The intelligent vehicle is a complicated nonlinear system, and the design of a path tracking controller is one of the key technologies in intelligent vehicle research. This paper mainly designs a lateral control dynamic model of the intelligent vehicle, which is used for lateral tracking control. Firstly, the vehicle dynamics model (i.e., transfer function) is established according to the vehicle parameters. Secondly, according to the vehicle steering control system and the CARMA (Controlled Auto-Regression and Moving-Average) model, a second-order control system model is built. Using forgetting factor recursive least square estimation (FFRLS), the system parameters are identified. Finally, a neural network PID (Proportion Integral Derivative) controller is established for lateral path tracking control based on the vehicle model and the steering system model. Experimental simulation results show that the proposed model and algorithm have the high real-time and robustness in path tracing control. This provides a certain theoretical basis for intelligent vehicle autonomous navigation tracking control, and lays the foundation for the vertical and lateral coupling control.

## 1. Introduction

In recent years, intelligent vehicles play an important role in the intelligent transportation system. They have attracted considerable attention to the research community and industry. Different functionalities are already available in commercial vehicles, such as, early mature technology, the Antilock Braking (ABS) [1], Traction Control System (TCS) [2], Electronic Stability Program(ESP) [3], Electric Power Steering (EPS) [4,5], and Electronic Braking System (EBS) [6,7], and Automatic Braking System (ABS) [8]. There are also newer technologies such as adaptive cruise control system (ACC) [9], Automatic Parking System (APS) [10], Anti-Collision System (ACS) [11,12,13],and others Advanced Driver Assistance Systems (ADAS) [14,15,16,17].The ultimate goal of the technological development is to realize automatic driving.

Automatic driving is a complex process, and it is also a process of self-learning which includes environmental recognition, real time localization, path planning and motion tracking control [18,19,20,21,22]. In this case, soft computing techniques provide the advantage of representing expert knowledge for controlling complex and nonlinear processes, such as autonomous driving. Several control strategies have been developed in the literature: in reference [23], the output feedback self-tuning controller proposed for the vehicle lateral control problem is developed. $H_{\infty }$ controller [24] based on the loop shape procedure for control, and widely use adaptive tracking control system in references [25,26,27]. The PID control method is a typical representative of classical control algorithms, PID controllers were proposed in [28,29,30] and used these for the experimental evolution of control. Sliding mode controllers [31,32] are used for trajectory tracking. The fuzzy logic control [33] is used for a skid steering vehicle. The neural network controller is used for the steering vehicle in [34], and it is widely used in machine learning for nonlinear patterns [35,36].The above methods are independently used, and there are some shortcomings in terms of system control. Combining the above methods for the control system is more widely in practical application, and the control effect is better in [37,38,39,40,41]. Other techniques are too numerous to be listed here.

These methods consider the problem of trajectory tracking for nonlinear systems state change process. However, for intelligent vehicles at different speeds, the computation time (for optimization algorithm) becomes very difficult for real-time operation. Different methods showed that the class of adaptive controllers represents a very promising technique for such uncertain and nonlinear application [19].

The neural networks PID controller can achieve good results in controlling an intelligent vehicle, and provide safe driving. This control technique has proven to be robust against system parameter variations. Zhang et al. [40,41] applied to keep and track the pre-given trajectory in the lateral control. This control strategy is well suited for driving applications. In addition, an algorithm of vehicle lateral adaptive PID control with neural network was proposed. The parameters of PID control are tuned by back propagation neural networks. In the tuning process, the plant predictive output is used to modify the weights of neural networks; it is nonlinear prediction which improves the predictive accuracy.

This paper focuses on motion tracking control, which is mostly dedicated to the lateral control. Lateral control is concerned with steering the vehicle automatically in order to follow the reference path. First, the vehicle model is established according to the vehicle parameters. Second, according to the vehicle steering control system and the CARMA model, a second-order control system model is established, and system identification is used to adjust the model parameters. Finally, based on the previous research [42,43] in the path planning adopting behavior dynamics method, using the established neural network PID controller, the intelligent vehicle planning tracing controlling is realized.

This paper is organized as follows: Section 2 describes the analysis of vehicle model and dynamics. The vehicle steering system equivalent to the actual model and the model parameter identification is introduced in Section 3. In Section 4, the design of the lateral BP (Back Propagation) neural network PID controller of the autonomous vehicle for path tracking is presented; simulation results of illustration examples and discussion are also presented. Finally, the conclusion and further discussion is given in Section 5.

## 2. Vehicle Dynamics Analysis and Modeling

Vehicles can be treated as a multiple rigid body systems including components, motion pair, mechanical elements, etc. Vehicle model is a highly complicated nonlinear system; it is often difficult to use one or more of the mathematical formulas to accurately describe. Therefore, the dynamic model of vehicle should be simplified to derivate the model of vehicle in order to calculate and analyze its dynamic characteristic. This design uses a simplified vehicle model, which converts four-wheel vehicle models to two rounds of vehicle models [20].

### 2.1. Vehicle Model

**Figure 1:**
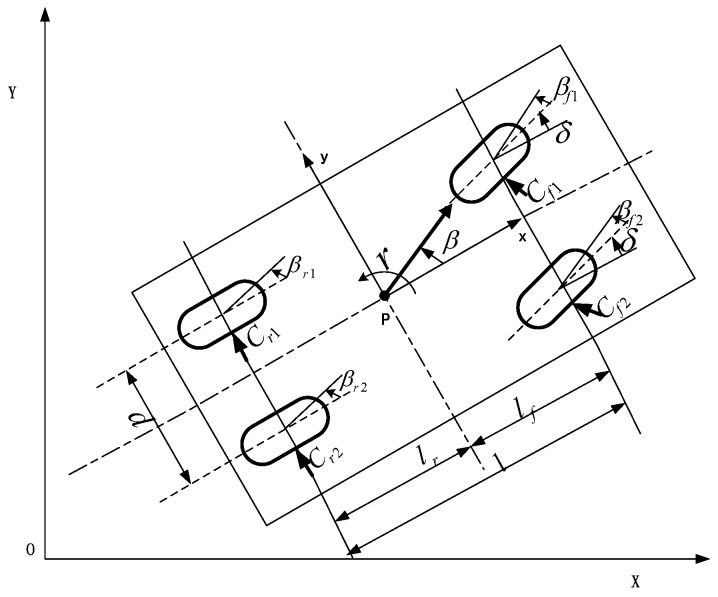
Four-wheel vehicle model.

**Figure 2:**
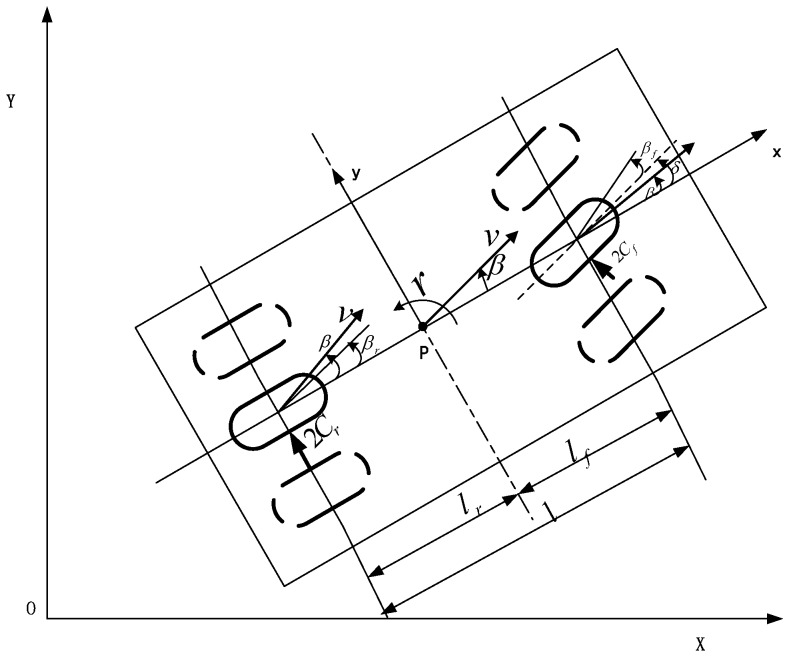
Two-wheel vehicle model.

The four-wheel vehicle model and two rounds of vehicle model are shown in Figure 1 and Figure 2.

**Figure 3:**
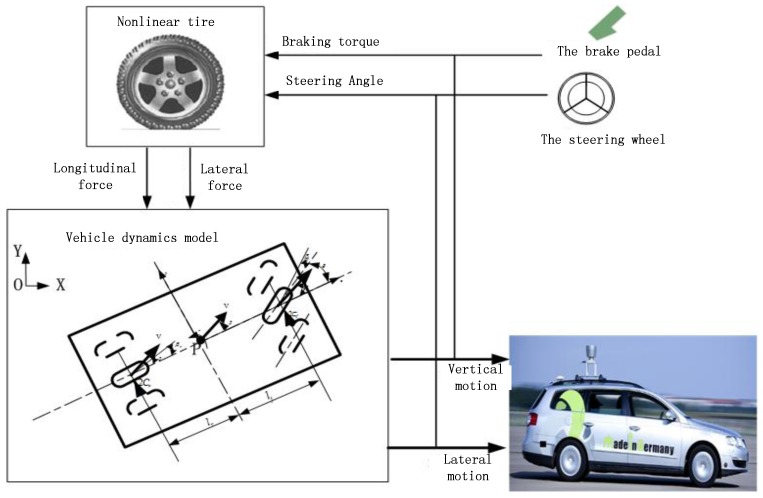
Vehicle system model.

According to the simplified two-wheel vehicle model, the vehicle system model is established, which is shown in Figure 3.

### 2.2. Vehicle Dynamics Analysis

According to the simplified two-wheel vehicle model for vehicle dynamics analysis, the two degrees of freedom vehicle kinematics equation is set up:

Side-slip Angle and yawing angular velocity:

{(1)mVxdβdt+μ(Kf+Kr)β+[mVx+μ(lfKf−lrKr)Vx]dψdt=μKfδ(2)Izd2ψdt2+μ(lfKf−lrKr)β+μ(lf2Kf+lr2Kr)Vxdψdt=μlfKfδ

In Equations (1) and (2) for Laplace transform: $\dot{\psi }=\omega$

(3) $$\left[\begin{array}{cc} mV_{x}s+\mu (K_{f}+K_{r}) & mV_{x}+\frac{2}{V_{x}}(l_{f}K_{f}+l_{r}K_{r})\\ \mu (l_{f}K_{f}+l_{r}K_{r}) & Is+\frac{2}{V_{x}}(l_{f}^{2}K_{f}+l_{r}^{2}K_{r}) \end{array}\right]\left[\begin{array}{c} \beta (s)\\ \omega (s) \end{array}\right]=\left[\begin{array}{c} \mu K_{f}\delta (s)\\ \mu l_{f}K_{f}\delta (s) \end{array}\right]$$

According to Equations (3) to (4):

(4) $$\frac{d^{3}\psi }{dt^{3}}+\mu (\frac{l_{f}^{2}K_{f}+l_{r}^{2}K_{r}}{V_{x}I_{z}}+\frac{K_{f}+K_{r}}{mV_{x}})\frac{d^{2}\psi }{dt^{2}}+(\mu \frac{l_{r}K_{r}-l_{f}K_{f}}{I_{z}}+\frac{K_{f}K_{r}L^{2}}{mV_{x}^{2}I_{z}})\frac{d\psi }{dt}=\frac{Ll_{f}K_{f}}{mV_{x}I_{z}}\frac{d\delta }{dt}+\frac{LK_{f}K_{r}}{mV_{x}I_{z}}\delta$$

a_0_, a_1_, b_0_, b_1_ are as follows:

$$\begin{array}{l} a_{0}=\mu (\frac{l_{f}^{2}K_{f}+l_{r}^{2}K_{r}}{V_{x}I_{z}}+\frac{K_{f}+K_{r}}{mV_{x}})\\ a_{1}=\mu \frac{l_{f}K_{f}-l_{r}K_{r}}{I_{z}}+\frac{K_{f}K_{r}L^{2}}{mV_{x}^{2}I_{z}} \end{array}\begin{array}{l} b_{0}=\frac{Ll_{f}K_{f}}{mV_{x}I_{z}}\\ b_{1}=\frac{LK_{f}K_{r}}{mV_{x}I_{z}} \end{array}$$

The Equation (4) is simplified to Equation (5):

(5) $$\dddot{\psi }+a_{1}\ddot{\psi }+a_{0}\dot{\psi }=b_{1}\dot{\delta }+b_{0}\delta$$

According to $\dot{\psi }=\omega$ available Equation (6):

(6) $$\frac{\omega (s)}{\delta (s)}=W_{1}(s)=\frac{b_{1}s+b_{0}}{s^{2}+a_{1}s+a_{0}}$$

Vehicle parameters are shown in Table 1.

According to the vehicle parameters and Equation (6), the specific transfer function can be obtained:

(7) $$W_{1}(s)=\frac{b_{1}s+b_{0}}{s^{2}+a_{1}s+a_{0}}=\frac{11.79s+43.12}{s^{2}+5.125s+6.814}$$

## 3. Vehicle Steering System Control Modeling

In the control system, it is necessary to find a system equivalent to the actual model in order to facilitate the practice test. Therefore, it is import that the model is chosen from a set of models, according to certain principles and the best fitting of the concern of dynamic or static characteristics of the actual system. The main tasks of Identification include determining model structure, the estimated model parameters and the unknown test model results [44,45].

### 3.1. Identification Signal 

In the intelligent vehicle system, the vehicle steering system is a slow change of a strongly nonlinear system, and this feature will often bring great difficulty in vehicle dynamics research. The steering system is mainly composed of a steering mechanism, steering gear and a transmission mechanism, etc., so the vehicle’s steering system can be at least a second order system model. The steering wheel angle with the number of input pulses is one-to-one correspondence. However, when the number of input pulses is the same, the steering wheel angle is different, with the noise of the mathematical model of the system given.

In engineering, generally, the longest linear shift register sequence (such as M sequence) is chosen as the identification input signal. Four order M sequences are used as input signals, which is the input voltage signal of the steering stepper motor controller in the actual physical significance.

### 3.2. Identification and Analysis of Vehicle Steering System

The vehicle steering system and driving system are graded as a strongly nonlinear system. When the vehicle is in normal operation, the vehicle steering system can be approximately described by a two order system. The vehicle steering system is described as the output signal of the steering servo motor, driven by the drive as the front wheel steering structure, and driven by the front wheel rotation. The transfer function is the relationship between the motor input pulse number and the lateral angular velocity.

In the experimental process, the model of identification of the vehicle steering system is the CARMA (Controlled Auto-Regression and Moving-Average) model of using the least squares identification algorithm. However, the ordinary least squares identification method can elicit the “data saturation” phenomenon and lose correction ability, and for the time-varying system, it will lead to the change of the parameter estimates as it cannot track the time-varying parameters. The forgetting factor recursive least squares algorithm (FFRLS) is used to estimate time-varying parameters. The transfer function of the steering system by the bilinear transformation can get its discrete form:

Based on the CARMA model of difference Equation (8):

(8) $$A(z^{-1})z(k)=B(z^{-1})u(k)+C(z^{-1})\varepsilon (k)$$

u(k) and z(k) are the input and output system, ε(k) is the mean to 0,for constant unrelated noise variance, $A(z^{-1})$ , $B(z^{-1})$ and $C(z^{-1})$ polynomials parameter. The CARMA model Equation (7) can be converted to the least square of parameter identification problems Equation (9):

(9) $$z(k)=\psi^{T}(k)\theta +\varepsilon (k)$$

The input and output value and unknown parameter vector θ , respectively.

(10) $$\{\begin{array}{c} {\hat{\psi }}^{T}(k)=[-z(k-1),\cdots ,-z(k-n_{a}),u(k-1),\cdots ,u(k-n_{b}),\varepsilon (k-1),\cdots ,\varepsilon (k-n_{c})]\\ \hat{\theta }=[{\hat{a}}_{1}\cdots {\hat{a}}_{n_{a}},{\hat{b}}_{0}\cdots {\hat{b}}_{n_{b}},\hat{c}1\cdots {\hat{c}}_{n_{c}}){]}^{T} \end{array}$$

Selection criterion function $J(\theta )=\frac{1}{2}E\{[z(k+n)-\psi^{T}(k+n)\theta ]\}$ , can get the parameters of the algorithm as shown in Equation (11).

(11) $$\hat{\theta }(k+n)=\hat{\theta }(k-1)+\rho (l)\psi (k+n)[z(k+n)-\psi^{T}(k+n)\hat{\theta }(k-l)]\begin{array}{c} k=1,2\cdots \end{array}$$

$$\{\begin{array}{c} \hat{\theta }(k)=\hat{\theta }(k-1)+K(k)[z(k)-{\hat{\psi }}^{T}(k)\hat{\theta }(k-1)]\\ K(k)=P(k-1)\hat{\psi }(k){\left[\mu +{\hat{\psi }}^{T}(k)P(k-1)\hat{\psi }(k)\right]}^{-1}\\ P(k)=\frac{1}{\mu }[I-K(k){\hat{\psi }}^{T}(k)]P(k-1)\\ 0<\mu \leq 1 \end{array}$$

According to the above theory, under the CARMA model, the establishment of an intelligent vehicle steering model system of second order differential Equation (12):

(12) $$z(k)=a_{1}z(k-1)+a_{2}z(k-2)+b_{0}u(k-1)+b_{1}u(k-2)+\varepsilon (k)+c_{1}\varepsilon (k-1)+c_{2}\varepsilon (k-2)$$

$${a=[1-1.795\;0.8972]}^{\prime }\;{b=[2.625\times 10^{-6}]}^{\prime }\;a={\left[1\;0.3\;0.2\right]}^{\prime }$$

### 3.3. Identification of Simulation Calculation

**Figure 4:**
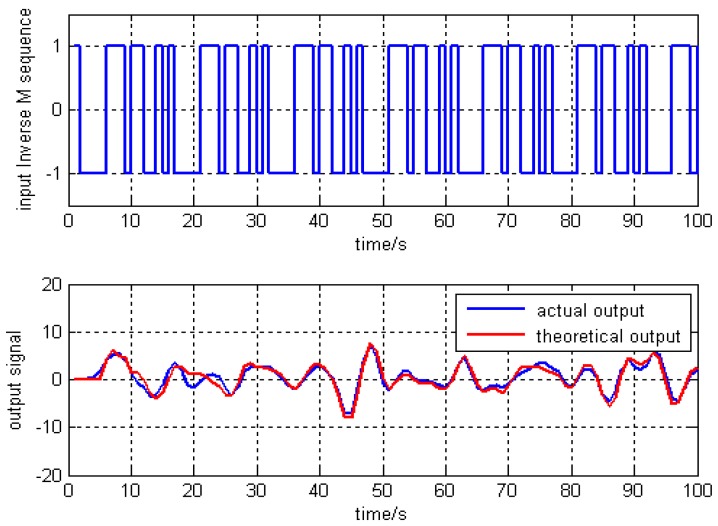
Response curve of angular frequency of steering wheel.

Using the above algorithm identification, there was a sampling time of 0.01 s, the number of iterations is 100 times. The fourth-order inverse M sequence input signal, and the simulation output of steering wheel Angle and the actual output are shown in Figure 4.

**Figure 5:**
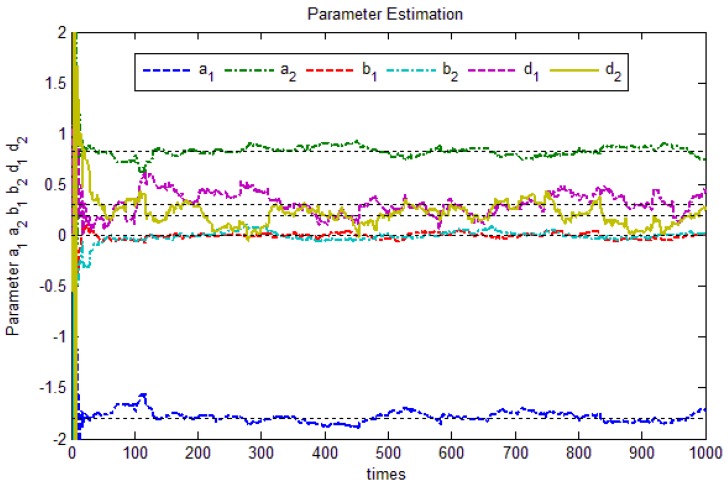
The parameter estimation process.

Using forgetting factor recursive least squares estimation (FFRLS), the forgetting factor value is 0.98, the theoretical value of equations of each factor is $\hat{\theta }\;(0)\;=\;[\begin{array}{cccccc} 0 & 0 & 0 & 0 & 0 & 0 \end{array}{]}^{T}$ , $P(0)\;=\;10^{6}\;\times \;I_{6\times 6}$ . FFRLS algorithm is adopted to the parameter estimation, system parameter estimation process is shown in Figure 5, When K=1000, ${\hat{a}}_{1}=-1.79559$ , ${\hat{a}}_{2}=0.84126$ , ${\hat{b}}_{1}=0.000654$ , ${\hat{b}}_{2}=0.000354$ , ${\hat{d}}_{1}=0.202272$ , ${\hat{d}}_{2}=0.27674$ .

In Figure 5, *d*_1_ and *d*_2_ parameters converge slower, and other parameters converge, which is caused by inaccuracy in the white noise estimation. In practice, however, increasing the simulation steps can improve the parameter estimation accuracy.

**Figure 6:**
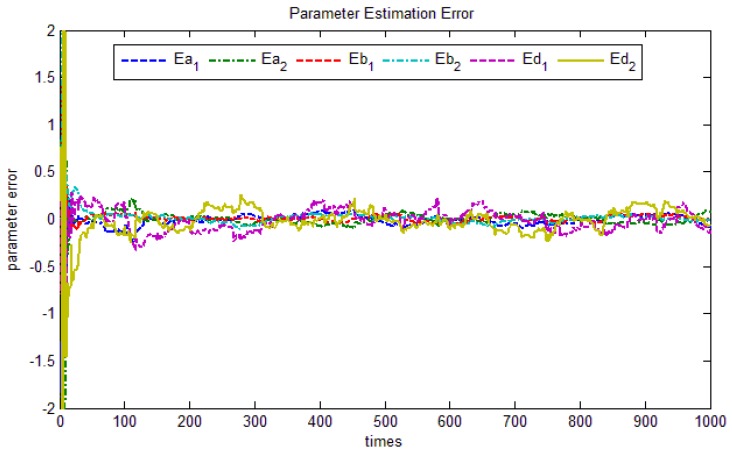
The parameter estimates convergence.

The parameter error is shown in Figure 6, the parameters estimation error of d_1_ and d_2_also exhibit slower convergence, which is caused by inaccurate white noise estimation. The other parameter error tends to zero finally. Thus, we get the transfer function Equations (13) and (14):

(13) $$H(z)=\frac{{\hat{b}}_{2}z+b_{1}}{z^{2}+{\hat{a}}_{2}z+{\hat{a}}_{1}}=\frac{0.0002399z+0.0008576}{z^{2}-1.716z+0.7596}$$

(14) $$W_{2}(s)=\frac{-0.0008623s^{2}-0.09355s+53.2}{s^{2}+19.02s+378.2}$$

Equations (7) and (13) are connected as a controlled object.

## 4. The Heading Angle Neural Network PID Control System

### 4.1. The Neural Network PID Control Structure

PID controller is a feedback loop in industrial control applications. The collected data and a reference value are compared to input of the controller; the error value is used to calculate the new input values. This experiment proves that other control methods may cause the system to create unstable error or shocks, and PID feedback loop can retain the stability of the system.

**Figure 7:**
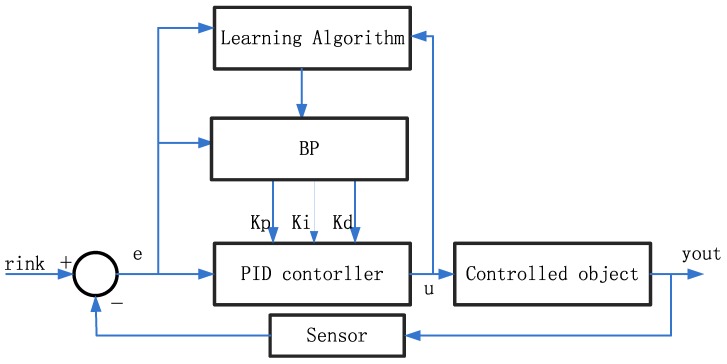
BP neural network PID control system.

The BP (Back-Propagation) neural network can approximate arbitrary nonlinear systems and the structure and learning algorithm are simple. Through neural network self-learning ability, Kp, Ki, Kd parameters can find an optimal control law. Based on the BP neural network, the PID (Proportion Integral Derivative) controls system structure is shown in Figure 7:

The controller is described using the incremental PID control algorithm and three layer neural network. The PID control equation is as follows Equation (15):

(15) $$\begin{array}{c} u(k)=u(k-1)+\Delta u(k)\\ \Delta u(k)=K\sum_{i=1}^{3}w_{i}(k)x_{i}(k)=K(w_{1}(k)x_{1}(k)+w_{2}(k)x_{2}(k)+w_{3}(k)x_{3}(k))\\ x_{1}(k)=e(k)-e(k-1),x_{2}(k)=e(k),x_{3}(k)=e(k)-2e(k-1)+e(k-2)\\ Kp=w_{1}(k),Ki=w_{2}(k),Kd=w_{3}(k) \end{array}$$

Kp, Ki, Kd is the proportion, integral, differential coefficient.

The control algorithm is summarized as follows:

- Step1: The determined system structure of the BP network is 1-3-1, the given system weight coefficients initial value, learning rate and inertia coefficient, the iteration times *k* = 1.
- Step2: Sampling get rink(k) and yout(k), calculating error at time: e(k) = rink(k) − yout(k).
- Step3: According to calculate input and output of NN neurons in each layer by, the output of the NN is PID controller parameters K_p_, K_i_, K_d_.
- Step4: According to the Equation (15) to calculate the output of the PID controller u(k) .
- Step5: Neural network learning, online adjust the weighting coefficient to realize the adaptive adjustment of PID control parameters.
- Step6: The iteration times k = k + 1, to return Step 2.

### 4.2. The Heading Angular Control

**Figure 8:**
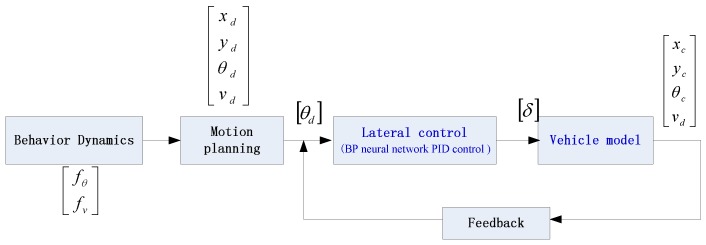
The control system model.

To verify the effectiveness of the proposed control algorithms, let us consider a two-wheeled vehicle as shown in Figure 2. In the simulation, the vehicle parameters are given Table 1. Moreover, in the neural network PID closed-loop system, two neural network PID controllers are used to control the longitudinal and angular subsystem separately, and the separated subsystems are of the second-order form independently. Then, the neural network PID controller can be changed to a single-input single-output (SISO) direct estimator. The following shows the test simulation model and controller. The experimental testing process is implemented according to Figure 8.

### 4.3. Adaptive PID Neural Network Controller Stability Analysis

A mathematical model of adaptive PID neural network controller is above the Equation (15):

Through the Lyapunov [46] stability analysis is as follows Equation (16):

(16) $$v(k)=\frac{1}{2}\sum_{i=1}^{k}e^{2}(i)$$

The BP neural network learning process leads to the change of v(k) as follows:

(17) $$\Delta v(k)=\frac{1}{2}(\sum_{i=1}^{k+1}e^{2}(i)-\sum_{i=1}^{k}e^{2}(i))=\frac{1}{2}\sum_{i=0}^{k}\left(e^{2}(i+1\right)-e^{2}(i))$$

If e(0)=0 , then

(18) $$\Delta v(k)=\frac{1}{2}\sum_{i=0}^{k}\left((e(i\right)+\Delta e(i){)}^{2}-e^{2}(i))=\frac{1}{2}\sum_{i=0}^{k}\left(2e(i\right)\Delta e(i)+\Delta e^{2}(i))$$

Due to BP the learning process, the change of the error is Equation (19):

(19) $$e(k+1)=e(k)+\Delta e(k)=e(k)+{\left(\frac{\partial e(k)}{\partial w(k)}\right)}^{T}\Delta w(k)$$

The objective function is Equation (20):

(20) $$J(k)=\frac{1}{2}\sum_{i=1}^{k}{\left(r(i)-c(i)\right)}^{2}=\frac{1}{2}\sum_{i=1}^{k}e^{2}(i)$$

In order to J(k) and w(k) is the negative gradient direction, we have the following Equation (21):

(21) $$w(k+1)=w(k)-Ζ(\frac{\partial J(k)}{\partial w(k)})$$

By Equations (20) and (21), Equation (22) can be obtained:

(22) $$\frac{\partial J(k)}{\partial w(k)}=e(k)\frac{\partial J(k)}{\partial \Delta u(k)}\frac{\partial u(k)}{\partial w(k)}$$

$$\Delta w(k)=-Ζ\frac{\partial J(k)}{\partial w(k)}=-Ze(k)\frac{\partial e(k)}{\partial \Delta u(k)}\frac{\partial \Delta u(k)}{\partial w(k)}$$

If

$$M={\left[\frac{\partial e(k)}{\partial w(k)}\right]}^{T}=\frac{\partial e(k)}{\partial \Delta u(k)}{\left[\frac{\partial \Delta u(k)}{\partial w(k)}\right]}^{T}$$

then,

(23) $$\Delta e(k)=-ΖMM^{T}e(k)$$

By bringing Equation (23) to (17), Equation (24) is derived:

(24) $$\begin{array}{cc} \Delta v(k) & =\frac{1}{2}\sum_{i=0}^{k}\left(-2e(i\right)ZMM^{T}{e(i)}^{T}+Z^{2}MM^{T}(M^{T}{e(i)}^{T}A^{T}e(i))\\ & =-\frac{1}{2}\sum_{i=0}^{k}(A^{T}e{\left(i\right)}^{T}(2Z-Z^{2}MM^{T})(M^{T}e(i))) \end{array}$$

Based on the Lyapunov stability theory, when Δv(k)<0 , the whole control system is stable, so $2Z-Z^{2}MM^{T}>0$ , get in range of $0<Z<2{\left(MM^{T}\right)}^{-1}$ . Due to Δv(k)<0 , get in $1/2e^{2}(k+1)<1/2e^{2}(k)$ , $\underset{k\to \infty }{\lim}e(k)=0$ . With the increase of k, the e(k) is tend to zero, the learning algorithm convergence. From what has been discussed above, the effect of the adaptive PID neural network algorithm has a relation with the selection of the adjustable parameters K(xiteP, xiteI, xiteD), Kp, Ki, Kd, etc.

### 4.4.Simulation Experiments

#### 4.4.1. Tracking the Curve

**Figure 9:**
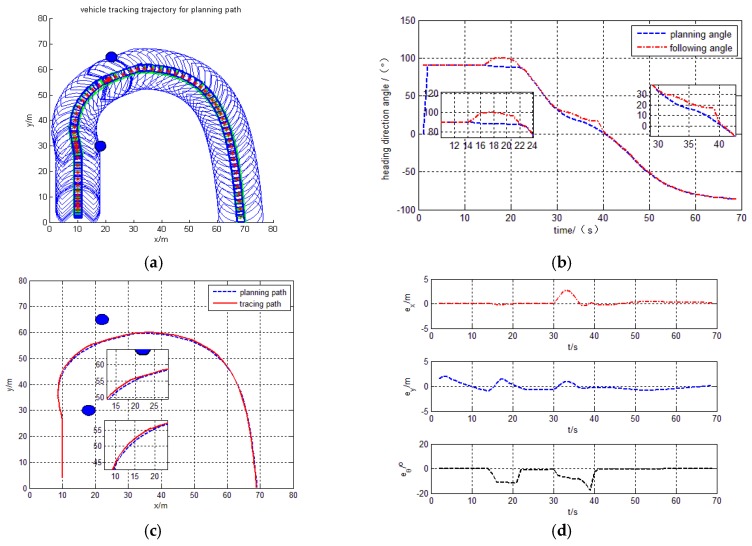
The curve behavior tracking trajectory with barrier. (**a**) Path tracing trajectory; (**b**) Heading direction angle; (**c**) Planning path and tracing path; (**d**) X and Y direction and heading angle error.

First, suppose that the vehicle tracks a curve trajectory. In this paper, MATLAB R2013a simulation experiments are shown in Figure 9. The simulation parameters: planning path start point is (10, 5, 0, 1.4) (initial x, y position (m), heading angle (°), speed(m/s)), tracing vehicle initialization parameters for followVehiclePos = (10,3,90,1,8,90,1).Besides, the BP parameters are chosen as follows: xiteP = 0.40, xiteI = 0.35, xiteD = 0.40, the PID parameters are set Kp = 0.10, Ki_1 = 0.10, Kd_1 = 0.10.

**Figure 10:**
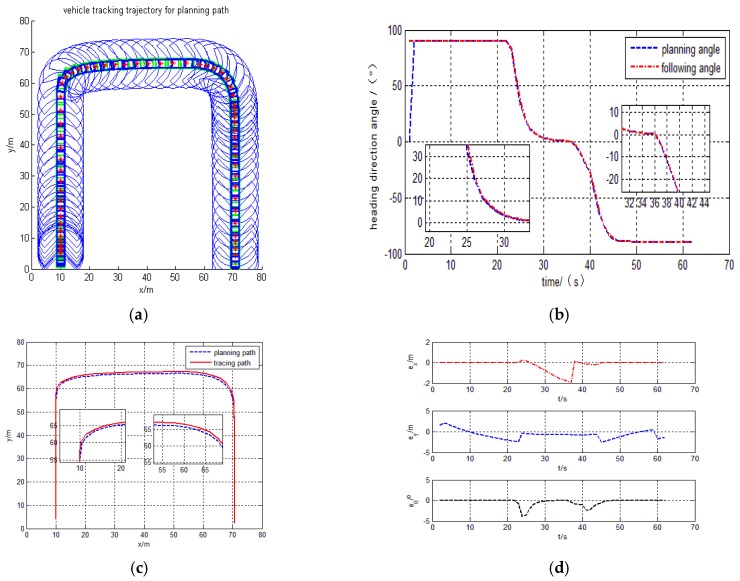
The curve behavior tracking trajectory without barrier.(**a**) Path tracing trajectory; (**b**) Heading direction angle; (**c**) Planning path and tracing path; (**d**) X and Y direction and heading angle error.

With these design parameters, the practical tracking trajectory for the curve trajectory is in Figure 10a, and the heading direction angle is shown in Figure 10b.The tracing curve point is in Figure 10c, X direction and Y direction error is also shown in Figure 10d.

The simulation results deduce that the intelligent vehicle can track planning trajectory very well with the proposed design control model. It observes that there exists a drastic regulation process on the turning place, because heading direction angle change is larger on the turning place and results upright for vehicle body. However, from the partially enlarged drawing in Figure 10c, the tracking errors $e_{x}$ and $e_{y}$ are explicitly demonstrated to an acceptable bound within ±2 m, and the tracking heading angle errors $e_{\theta }$ is ±3°, they are also shown in Figure 10d.

Judging by the simulation results, the intelligent vehicle can track planning trajectory very well with a barrier using the proposed design control model. The trajectory, tracking and heading angle of the intelligent vehicle are also shown in Figure 9a–c. The tracking errors $e_{x}$ , $e_{y}$ and the heading errors $e_{\theta }$ are also shown in Figure 9d. The experimental results show that the method is more general.

#### 4.4.2. Tracking the Overtaking Behavior

**Figure 11:**
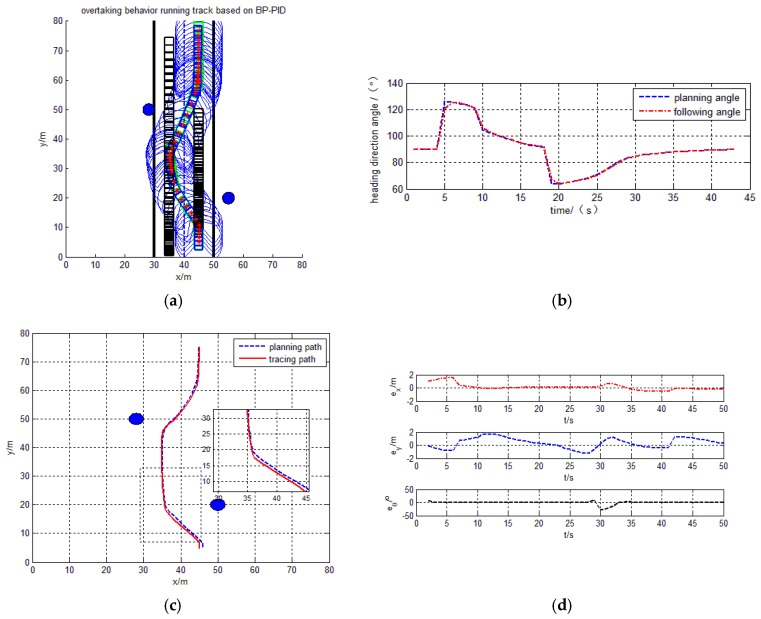
Overtaking behavior BP-PID tracing trajectory. (**a**) Path tracing trajectory; (**b**) heading direction angle; (**c**) Planning path and tracing path; (**d**) X and Y direction and heading angle error.

**Figure 12:**
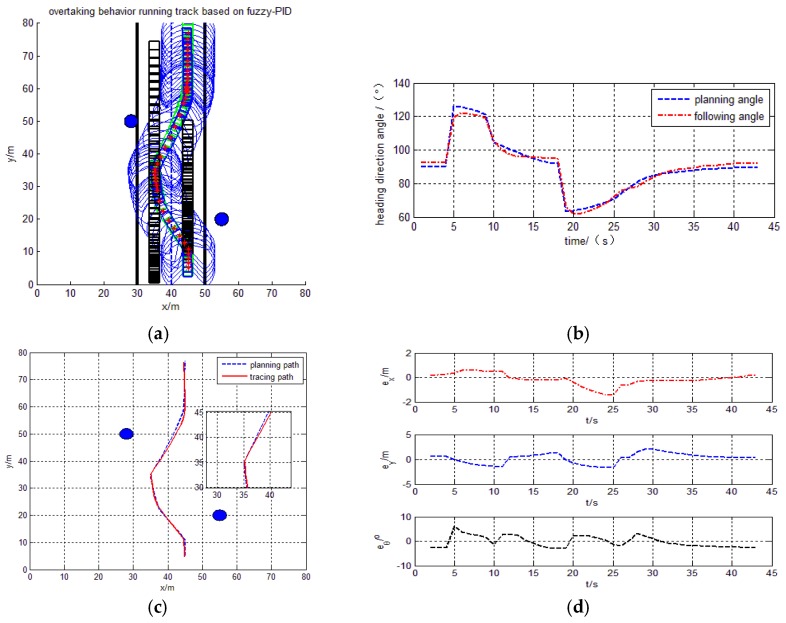
Overtaking behavior Fuzzy-PID tracing trajectory. (**a**) Path tracing trajectory; (**b**) Heading direction angle; (**c**) Planning path and tracing path; (**d**) X and Y direction and heading angle error.

By the simulation results, the intelligent vehicle can track planning trajectory based on overtaking behavior using the proposed design control model in Figure 11. In Figure 12, the intelligent vehicle can track planning trajectory based on overtaking behavior using the Fuzzy-PID model. The trajectory, tracking and heading angle of the intelligent vehicle are also shown in Figure 11a–c and Figure 12a–c. The tracking errors $e_{x}$ , $e_{y}$ and the heading errors $e_{\theta }$ are also shown in Figure 11d and Figure 12d. The experimental results show smaller error, smoother for heading angle changes, and there is no appearance of a sharp turn.

## 5. Conclusions

This work designs a lateral control dynamic model of the intelligent vehicle, which is used for lateral tracking control. This control model comprises PID control for heading angle and BP Neural network control for the PID parameters adjustment. Experiments show that this method can effectively adapt to the dynamic road environments, and at the same time can be dynamically adjusted to the heading angle of the sharp change process according to the planning path, and the comfort of the vehicle lateral control process can be improved. Compared to the classic fuzzy PID in the process of the dynamic path tracking, the improved model has better robustness. The research results are obtained to demonstrate:

(1) The results of the pre-study behavioral dynamics motion planning are applied to the current motion tracking controller.
(2) The model of intelligent vehicle steering system is built by using CARMA model and the parameters of steering system is trained by using FFRLS identify method. The vehicle model is set up according to the parameters of intelligent vehicle. The vehicle steering system model and vehicle model is connected to estimable a second-order control system.
(3) The planning of the heading angle is input to the designed controller and output practices heading angle. An error between the planning path and tracing trajectory is calculated before feedback to the controller. The controller calculated the tracing heading angle in order to achieve zero path error.
(4) The experimental results show that the identification algorithm and the BP neural network PID control model have real-time performance and reliability in path tracing, and the heading direction angle tracking effect is good; x, y direction and heading angle error is controllable and is close to zero. The method will lay a foundation for the lateral and the longitudinal coupling control.

## Figures and Tables

**Table 1:** Vehicle parameters.

Sign	Meaning	Value	Unit
L × D × H	Vehicle size	3600 × 1600 × 1700	mm × mm × mm
μ	Road friction coefficient	2	
m	Vehicle Mass	1100	kg
$I_{z}$	Yaw moment of inertia	2850	kg m^2^
$l_{f}$	Front axle-COG distance	1.15	m
$l_{r}$	Rear axle-COG distance	1.05	m
$K_{f}$	Cornering stiffness of the front tire	32000	N/rad
$K_{r}$	Cornering stiffness of the real tire	32000	N/rad
v	Vehicle	≤60	km/h
